# Efficacy and safety of ESR-EB and ESE in the treatment of small gastric muscularis propria tumours: single-centre prospective cohort study

**DOI:** 10.1038/s41598-025-29949-5

**Published:** 2025-12-08

**Authors:** Zhaohui Liu, Chong Chen, Wanqing Zhang, Haijian Guo, Dayong Sun, Ruinuan Wu

**Affiliations:** 1 Departments of Gastroenterology, Shenzhen Second People’s Hospital, First Affiliated Hospital of Shenzhen University Health Science Center, Shenzhen, China; 2 Department of Pathology, Shenzhen Second People’s Hospital, First Affiliated Hospital of Shenzhen University Health Science Center, Shenzhen, 518035 China

**Keywords:** Endoscopic snare resection with an elastic band, Endoscopic submucosal excavation, Gastric muscularis propria tumour, Efficacy, Safety, Cancer, Diseases, Gastroenterology, Medical research, Oncology

## Abstract

Objective To compare the efficacy and safety of endoscopic snare resection with an elastic band (ESR-EB) and endoscopic submucosal excavation (ESE) for the treatment of gastric muscularis propria tumours ≤ 10 mm in size. Methods From April 2023 to October 2024, gastric muscularis propria tumours ≤ 10 mm in size that were resected via ESR-EB or ESE were prospectively collected at Shenzhen Second People’s Hospital. The general clinical characteristics, tumour location, tumour size, growth pattern, histological diagnosis, operation time, resection time, complete resection rate, incidence of intraoperative complications, postoperative antibiotic usage rate, postoperative hospital stay, follow-up time, and presence of recurrence and metastasis were compared between the two groups. Results A total of 245 patients were enrolled, 14 of whom were excluded due to having multiple muscularis propria tumours in the stomach. Therefore, 231 patients were ultimately included for analysis (108 patients in the ESR-EB group and 123 patients in the ESE group). There were no differences in sex or tumour growth pattern, but there were significant differences in age, tumour size and tumour location (P < 0.05). Propensity score matching (PSM) was used, resulting in 54 patients in each group. The operation time was significantly shorter in the ESR-EB group than in the ESE group (21.61 ± 9.31 min vs. 33.15 ± 19.00 min; P < 0.001). The resection time significantly shorter in the ESE-EB group than in the ESE group (9.85 ± 6.09 min vs. 26.39 ± 18.16 min; P < 0.001). A 100% complete resection rate was achieved in both groups. There was no significant difference in postoperative hospital stay between the two groups (5.81 ± 1.41 d vs. 5.39 ± 1.53 d; P = 0.161). GISTs represented the most common histological diagnosis in both groups. Thirty-two patients (59.26%) in the ESR-EB group had gastrointestinal stromal tumours (GISTs), and 27 patients (50.00%) in the ESE group had GISTs. The second most common histological diagnosis was leiomyoma, and schwannoma was the rarest histological diagnosis. There was no significant difference between the two groups in terms of histological diagnoses (P = 0.463). Perforation was the most common intraoperative complication, affecting 30 patients (55.56%) in the ESR-EB group and 21 patients (38.89%) in the ESE group; this difference was not significant (P = 0.083). Five patients (9.26%) in the ESR-EB group experienced intraoperative bleeding, which was significantly lower than the 18 patients (33.33%) in the ESE group (P = 0.002). All perforations and bleeding were successfully managed endoscopically. Twenty-six (48.15%) patients in the ESR-EB group and 17 (31.48%) patients in the ESE group used postoperative antibiotics; this difference was not significant (P = 0.077). There was no significant difference in follow-up time between the ESR-EB and ESE groups (240.50 ± 57.14 d vs. 238.41 ± 57.48 d; P = 0.054). Neither group experienced recurrence or metastasis during the follow-up period. Conclusion Both ESR-EB and ESE are effective and safe methods for the resection of gastric muscularis propria tumours. However, ESR-EB has a low incidence of intraoperative bleeding as well as short operation and resection times; thus, ESR-EB is a safer and time-saving endoscopic technique. Trial registration :This trial was registered at chictr.org.cn under identifier Chictr2300072856.

## Introduction

Gastrointestinal stromal tumours (GISTs) are the most common type of tumour in the gastric muscularis propria and are less than 10 mm in diameter. A previous study revealed that in patients with primary small GISTs, 49.6% of the lesions were ≤ 10 mm in size^1^. In a study of 98 autopsies by Agaimy, the prevalence of small GISTs was approximately 22.4%^2^. In a study of the natural progression of subepithelial tumours in the stomach, 450 (45.5%) of the lesions with a diameter < 10 mm were found to show an increasing trend of 29 (6.4%), and the rate of increase was 0.14 mm/month. The rate of increase in lesions smaller than 10 mm was 1.31 ± 1.38 mm/year^3^. In a study of 936 patients with GISTs ≤ 2 cm, 2 patients with GISTs ≤ 1 cm were found to have a pathologically intermediate or high risk postoperatively^4^. These results indicate that GIST is the most common tumour in the gastric muscularis propria and that the tumour size tends to increase. Even GISTs ≤ 10 mm can be classified as intermediate or high risk. Therefore, endoscopic resection of gastric muscularis propria tumours less than 10 mm in diameter is necessary.

The Expert Consensus on Endoscopic Diagnosis and Treatment of Submucosal Tumours in the Digestive Tract of China (2023 edition) recommends the use of endoscopic snare excision (ESE) or endoscopic full-thickness resection (EFTR) for the resection of lesions originating from the gastric muscularis propria^5^. ESE is recommended for the removal of lesions in the mucosal or submucosal layer. In clinical practice, it is difficult to remove tumours in the gastric muscular layer of ≤ 10 mm via ESE or EFTR due to the following reasons^6–13^: (1) the tumour is not clearly located after the mucosal incision; (2) the bleeding rate is high during the operation; and (3) the exposure time of the perforation is long, thereby increasing the risk of abdominal infection. Our preliminary research results revealed that endoscopic snare resection with an elastic band (ESR-EB) can be used to effectively remove tumours in the gastric muscularis propria due to its advantages of a short operation time and a low incidence of intraoperative adverse events^14,15^. However, no previous studies have compared ESR-EB and ESE for the treatment of gastric muscularis propria tumours less than 10 mm in diameter. Therefore, this single-centre, prospective cohort study compared the safety and efficacy of ESR-EB and ESE to provide evidence-based medical recommendations for the endoscopic treatment of gastric muscularis propria tumours.

## Methods

### Study design

**Fig. 1:**
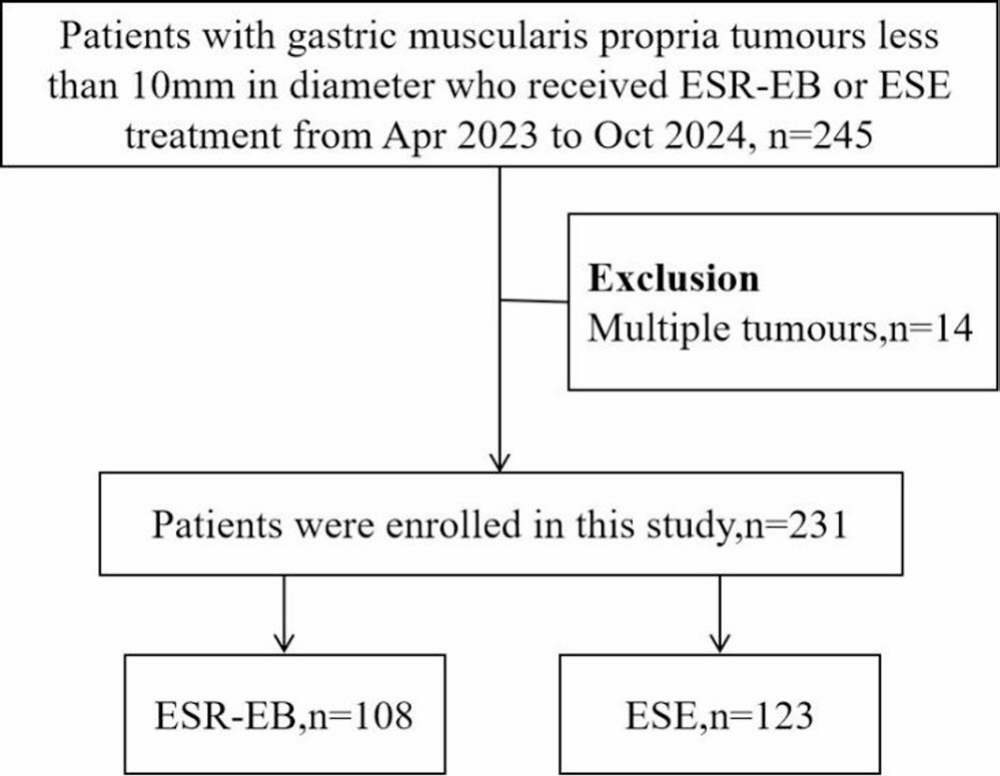
Flow chart of the patients enrolled in this study. ESR-EB, endoscopic snare resection with an elastic band; ESE, endoscopic submucosal excavation.

This single-centre, prospective cohort study was conducted at Shenzhen Second People’s Hospital. Enrolment occurred from April 2023 to October 2024. A total of 245 eligible patients were treated with ESE or ESR-EB; among them, 14 patients had multiple tumours in the gastric muscularis propria and were excluded. Ultimately, 231 patients were included in this study, including 108 patients in the ESR-EB group and 123 patients in the ESE group. This study was conducted in accordance with the 2008 revised Helsinki Declaration and approved by the Ethics Committee of Shenzhen Second People’s Hospital (2023-091-01PJ). All patients provided informed consent for endoscopic resection. The endoscopic removal method was determined by the endoscopist on the basis of the patient’s actual condition and personal preferences. The flowchart used for grouping is shown in Fig. 1.

### Patient selection criteria

The indications for ESR-EB and ESE were as follows: (1) for gastric tumours with a diameter ≤ 10 mm in the muscularis propria, endoscopic ultrasound (EUS) was used to measure tumour size; (2) computed tomography (CT) scans was performed to confirm the lack of lymph node involvement or distant metastasis; (3) patients were in stable condition without severe cardiopulmonary insufficiency and could tolerate endoscopic treatment; and (4) patients expressed willingness for endoscopic intervention. Eligible patients were considered suitable for ESR-EB or ESE procedures. The endoscopic resection method was selected according to the operator’s experience.

### Instruments

The following instruments were used: an endoscopic image processor (Olympus, Japan, CLV-290SL); a therapeutic endoscope (Olympus, Japan, HQ260J); an endoscopic ligation device (Boston Scientific, USA, M00542251); a high-frequency electrosurgical generator (ERBE, Germany, VIO300D); a snare (Boston Scientific, USA, M00561231; monofilament, 20 mm); a tissue clamp (Nanjing Minchuang Medical Technology Co., Ltd., China, POCC-D-26–195); and a disposable mucosal incision knife (Nanjing Minchuang Medical Technology Co., Ltd., China, KD-655 L).

### Intervention

#### ESR-EB procedure

**Fig. 2:**
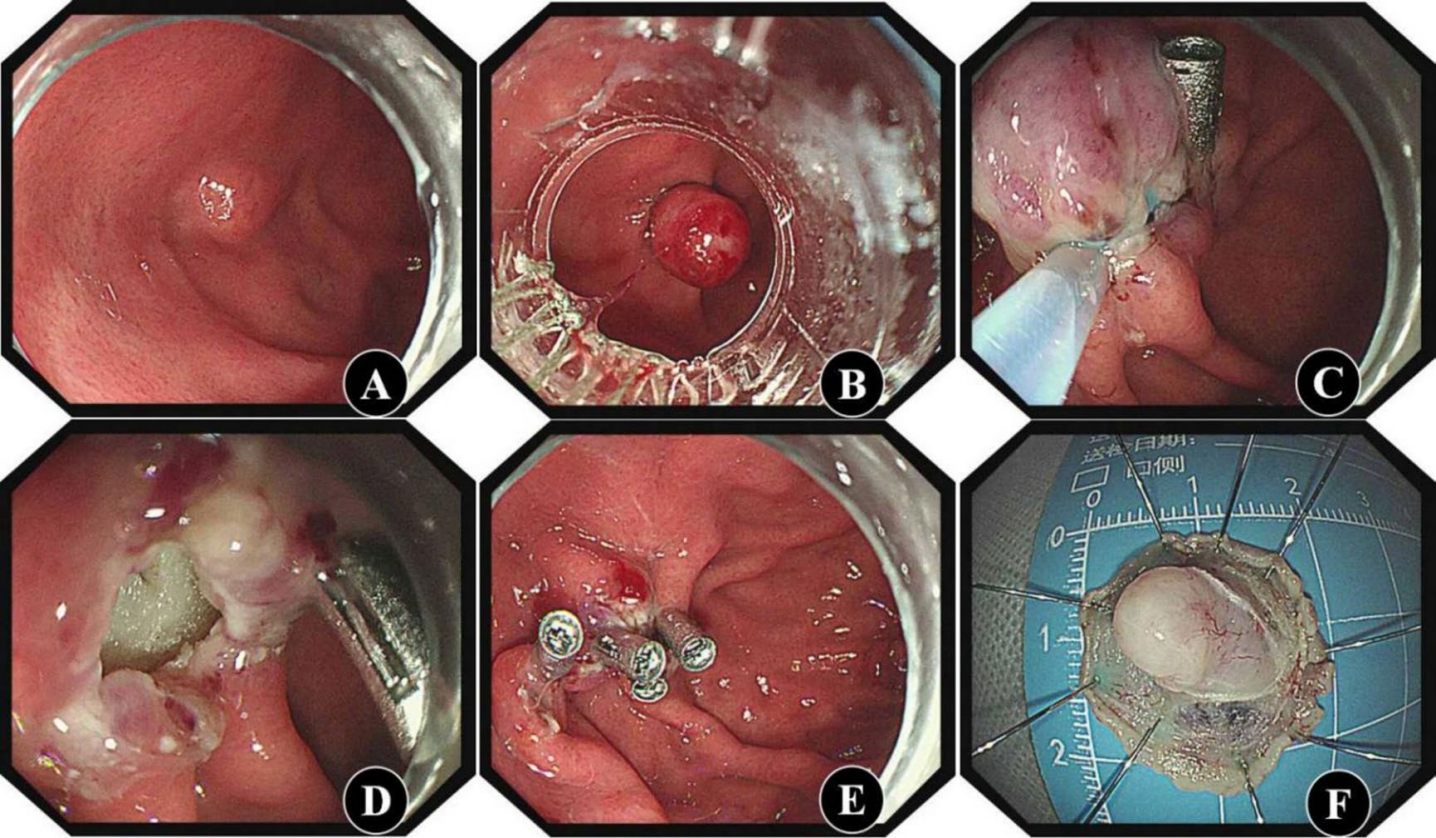
ESR-EB procedure** A**: The tumour was found by white light endoscope; **B**: The tumour was ligated with an elastic band; **C**: The snare was placed under the elastic band for removal; **D**: The wound was observed after excision; **E**: The wound was closed with clips; **F**: The excised specimen.

 A ligation device was installed at the front end of the gastroscope. The lesion was found in the gastric cavity (Fig. 2A). After the transparent cap was pressed against the lesion, negative pressure suction was used to completely inhale the lesion into the transparent cap, and the elastic band was released for ligation (Fig. 2B). The snare was placed under the elastic band and connected to an electric generator for slow electrocutting (Fig. 2C). We observed whether there was perforation or bleeding in the wound (Fig. 2D). If there was bleeding or a suspected vascular stump, electrocoagulation with a haemostat was performed. The wound was closed with clips (Fig. 2E). The resected lesion was removed from the body (Fig. 2F).

#### ESE procedure

**Fig. 3:**
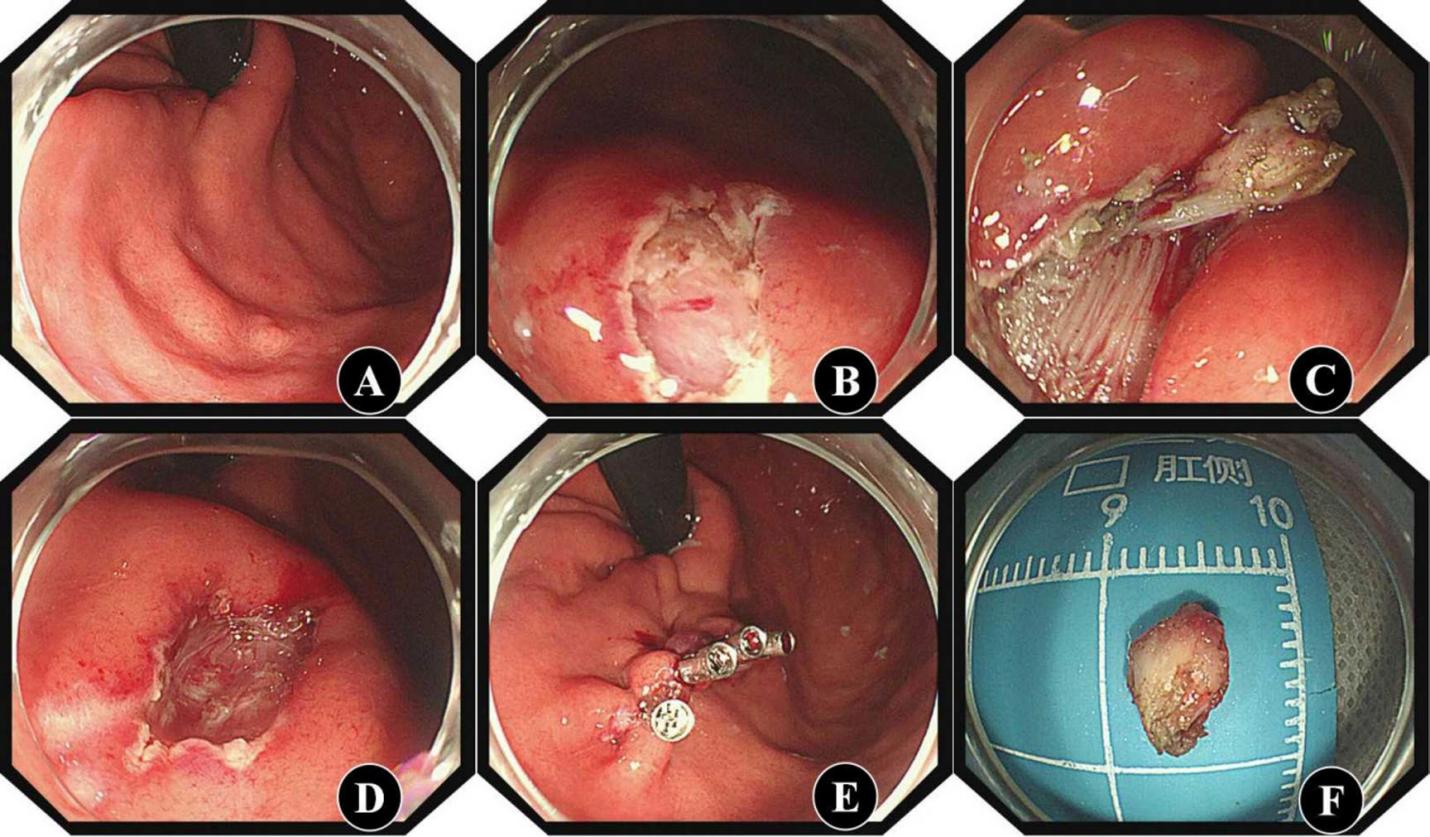
ESE procedure** A**: The tumour was found by white light endoscopy; **B**: The surface mucosa of the tumour was incised. **C**: The tumour was generally separated. **D**: The wound was observed after excision. **E**: The wound was closed with clips. **F**: The excised specimen.

The lesion was found in the gastric cavity (Fig. 3A). The surface mucosa of the tumour was incised with a disposable mucosal incision knife to expose the tumour (Fig. 3B). The tumour was separated along the lateral side to avoid damage to the tumour and prevent residual tumour or incomplete resection (Fig. 3C). We observed whether there was perforation or bleeding in the wound (Fig. 3D). If there was bleeding or a suspected vascular stump, electrocoagulation with a haemostat was performed. The wound was closed with clips (Fig. 3E). The resected lesion was removed from the body (Fig. 3F).

#### Outcome measures

We analysed the endoscopic complete resection rate, operative time, resection time, complication rate (including bleeding, perforation and gas-related complications), postoperative hospitalization time, and postoperative antibiotic rate. Follow-up assessments were performed to observe recurrence and metastasis. The endoscopic complete resection rate was defined as follows: the tumour was removed once endoscopically, without any gross residual tumour, and the pathological histology was negative for the margin. The operative time is defined as the interval between the beginning of endoscope entry into the mouth and the endoscope exiting from the mouth. The ESR-EB resection time was defined as the time from the start of the lesion being covered by the transparent cap to the end of the clips suturing the wound. The ESE resection time was defined as the time from when the surface mucosa of the tumour was incised to the end of the clip used to suture the wound. Intraoperative haemorrhage was defined as bleeding or a blood spurt that required electrocoagulation for haemostasis. Intraoperative perforation was defined as the visibility of intra-abdominal tissues and organs or the visible omentum under endoscopic vision. Gas-related complications were defined as intraoperative abdominal puncture for gas drainage or postoperative abdominal pain with radiographic evidence of free gas below the diaphragm.Postoperative hospitalization was defined as the date of discharge minus the date of operation. The antibiotic utilization rate was calculated as the number of patients who used antibiotics after surgery/total number of patients in the group ×100%. Recurrence was defined as the presence of a new raised mass at the site of tumour resection during follow-up. Metastasis was defined as the detection of lymphatic or organ metastases by imaging during follow-up.

### Sample size calculations

Based on data from previous literature^6–13^ and the results of our preliminary study, the complete resection rate of ESR-EB (Endoscopic Submucosal Resection with Electrosurgical Blade) is 100%, while that of ESE (Endoscopic Submucosal Excision) is 89.97%. Generally, 1-β is set at 0.8 or 0.9; in this case, 1-β = 0.8 is used as an example, meaning there is an 80% probability of detecting the true difference between the two groups if it exists. Here, β represents the type II error probability, which refers to the probability of incorrectly concluding that there is no difference between the two groups when an actual difference does exist.Substitute into the formula:

$$n = \frac{{(Z_{{1 - \alpha /2}} \sqrt {2\overline{P} (1 - \overline{P} } ) + Z_{{1 - \beta }} \sqrt {p_{1} (1 - p_{1} ) + p_{2} (1 - p_{2} )} )^{2} }}{{(p_{1} - p_{2} )^{2} }}$$

After calculation, the required sample size (n) for each group is approximately 60 cases. Considering a 20% loss-to-follow-up rate, the final required sample size for enrollment is calculated as 60 ÷ 0.8 ≈ 75 cases. Therefore, in this study, each group needs to enroll at least 75 patients.

### Statistical analysis

All the data were statistically analysed by SPSS28. The measurement data were normally distributed and thus are expressed as x ± s, and the intergroup comparisons were carried out via t tests. The frequency was used to describe the count data, and the intergroup comparison was based on the χ^2^ test (or corrected χ^2^ test or Fisher’s exact probability method). To mitigate the risk of selection bias, we applied propensity score matching (PSM) method with five covariates (sex, age, tumour size, tumour location, and growth pattern) to match the modified ESR-EB and ESE groups 1:1. The threshold for statistical significance was *P* < 0.05.

## Results

### Patient demographic and tumour characteristics

Before PSM, a total of 231 patients were included, with 108 in the ESR-EB group and 123 in the ESE group. There were no significant differences between the two groups in terms of sex or growth pattern (*P* > 0.05); however, there were significant differences in age, tumour size and tumour location between the two groups (*P* < 0.05). After matching, there were no statistically significant differences in sex, age, tumour location, tumour size, or growth pattern between the two groups (*P* > 0.05). The demographic and tumour characteristics of the patients in both groups are presented in Table 1.

**Table 1:** Patient demographic and tumour characteristics.

Variable	Before matching				After matching				
	ESR-EB, *n* = 108	ESE, *n* = 123	t/χ^2^	*P* value	ESR-EB, *n* = 54		ESE, *n* = 54	t/χ^2^	*P* value
Age (years)	55.83 ± 9.27	52.86 ± 11.56	2.166	0.031^*^	54.28 ± 9.04		55.02 ± 10.53	0.392	0.696
gender, n (%)			0.204	0.652				0.687	0.407
Male	33(30.56)	41(33.33)			15(27.78)		19(35.19)		
Female	75(69.44)	82(66.67)			39(72.22)		35(64.81)		
Location, n (%)			58.616	0.000^**^				0.000	1.000
Body	30(27.78)	96(78.05)			30(55.56)		30(55.56)		
Fundus	78(72.22)	27(21.95)			24(44.44)		24(44.44)		
Tumour size, mm	6.07 ± 1.42	6.89 ± 1.63	4.075	0.000^**^	6.69 ± 1.35		6.62 ± 1.66	0.241	0.810
Growth pattern, n (%)			0.311	0.856				2.160	0.142
Intraluminal growth	96(88.89)	112(91.06)			48(88.89)		52(96.30)		
Extraluminal growth	10(9.26)	9(7.32)			6(11.11)		2(3.70)		
Mixed growth	2(1.85)	2(1.63)			-		-		

ESR-EB: Endoscopic snare resection with an elastic band; ESE: Endoscopic submucosal excavation. ^*^<0.05,^**^<0.01.

### Comparison of treatment results between the two groups

Compared with the ESE group, the ESR-EB group required a significantly shorter operation time (21.61 ± 9.31 min vs. 33.15 ± 19.00 min; *P* < 0.001). Similarly, the resection time was markedly shorter in the ESR-EB group (9.85 ± 6.09 min vs. 26.39 ± 18.16 min; *P* < 0.001). The complete resection rate was 100% in both groups. The postoperative hospital stay did not differ between the two groups (5.81 ± 1.41 d vs. 5.39 ± 1.53 d; *P* = 0.161).

GISTs were the predominant histological diagnosis in both groups, all of which were classified as very low risk. GISTs accounted for 32 of 54 tumours (59.26%) in the ESR-EB group and 27 of 54 tumours (50.00%) in the ESE group. Leiomyomas were the second most common histological diagnosis, whereas schwannomas were the least common histological diagnosis. The distributions of the histological subtypes were comparable between the groups (*P* = 0.463).

Perforation was the most common intraoperative adverse event, occurring in 30 patients (55.56%) in the ESR-EB group and 21 patients (38.89%) in the ESE group; this difference was not statistically significant (*P* = 0.083). Conversely, the prevalence of intraprocedural bleeding was significantly less common in the ESR-EB group [5 patients (9.26%) vs. 18 patients (33.33%); *P* = 0.002]. All perforations and bleeding episodes were successfully managed endoscopically.No gas-related complications occuerred in either group.

Prophylactic antibiotics were administered to 26 patients (48.15%) in the ESR-EB group and 17 patients (31.48%) in the ESE group (*P* = 0.077). The median follow-up durations were similar (240.50 ± 57.14 days vs. 238.41 ± 57.48 days; *P* = 0.054), and no patients experienced recurrence or metastasis during surveillance (Table 2).

**Table 2:** Comparison of treatment results between the two groups.

Variable	Before matching				After matching				
	ESR-EB, *n* = 108	ESE, *n* = 123	t/χ^2^	*P* value	ESR-EB, *n* = 54		ESE, *n* = 54	t/χ^2^	*P* value
Operation time, min	21.22 ± 10.39	32.89 ± 20.90	5.471	0.000^**^	21.61 ± 9.31		33.15 ± 19.00	4.358	0.000^**^
Resection time, min	9.02 ± 5.00	24.84 ± 16.60	10.060	0.000^**^	9.85 ± 6.09		26.39 ± 18.16	6.913	0.000^**^
Complete resection, n (%)	54 (100.00)	54 (100.00)			54 (100.00)		54 (100.00)		
Post-operative hospital stay, d	5.75 ± 1.27	5.21 ± 1.34	3.091	0.002^**^	5.81 ± 1.41		5.39 ± 1.53	1.421	0.161
Histology diagnosis			26.285	0.000^**^				1.540	0.463
GIST, n (%)	82(75.93)	53(43.09)			32(59.26)		27(50.00)		
Leiomyoma, n (%)	23(21.30	66(53.66)			20(37.04)		26(48.15)		
Schwannoglioma, n (%)	3(2.78)	4(3.25)			2(3.70)		1(1.85)		
Intra-operative perforation, n (%)	79 (73.15)	33 (26.83)	49.396	0.000^**^	30 (55.56)		21 (38.89)	3.009	0.083
Intra-operative blooding, n (%)	5 (4.63)	53(43.44)	45.762	0.000^**^	5 (9.26)		18 (33.33)	9.336	0.002^**^
gas-related complications, n (%)	1(0.93)	4(3.25)	7.824	0.001^**^	0(0.00)		0(0.00)	-	-
Prophylactic antibiotics, n (%)	47(43.52)	28(22.76)	11.297	0.001^**^	26(48.15)		17(31.48)	3.130	0.077
Follow-up duration, d	227.26 ± 63.29	231.25 ± 66.64	1.552	0.085	240.50 ± 57.14		238.41 ± 57.48	1.971	0.054
Local recurrence, n (%)	0 (0.00)	0 (0.00)	-	-	0 (0.00)		0 (0.00)	-	-
Metastatic recurrence, n (%)	0 (0.00)	0 (0.00)	-	-	0 (0.00)		0 (0.00)	-	-

ESR-EB: Endoscopic snare resection with an elastic band; ESE: Endoscopic submucosal excavation. ^*^<0.05,^**^<0.01

## Discussion

Subepithelial tumours originating from the muscularis propria of the stomach are frequently encountered in clinical practice; lesions ≤ 10 mm are predominantly GISTs. Although these tumours are small, previous longitudinal studies have demonstrated a gradual increase in size over time^3^, and a subset of GISTs ≤ 10 mm have been classified as intermediate- or high-risk based on established criteria^4,16–18^. These findings underscore the need for proactive management of diminutive gastric muscularis propria–derived tumours.

The 2023 Chinese Expert Consensus on the Endoscopic Management of Subepithelial Lesions of the Gastrointestinal Tract^5^ recommends ESE and EFTR as standard endoscopic therapies for gastric tumours originating from the muscularis propria. Our preliminary data indicate that ESR-EB is a simple, rapid, effective and safe means of resecting such lesions. Therefore, we designed a single-centre, prospective cohort study to rigorously compare the clinical performance of ESR-EB with that of guideline-endorsed ESE for the resection of gastric muscularis propria tumours ≤ 10 mm. By generating high-quality, evidence-based data, this investigation seeks to inform clinical decision-making and may lay the groundwork for future updates to existing guidelines.

Our results demonstrated that both the total operation time and resection time were significantly shorter in the ESR-EB group than in the ESE group (*P* < 0.001). Consistent findings were reported by He et al.^19^ and Yan Meng et al.^8^, who compared ESR-EB with ESD or EFTR and similarly reported a marked reduction in operative duration. This advantage is attributable to the streamlined workflow of ESR-EB, which circumvents the multistep sequence of mucosal incisions, tumour identification and circumferential dissection required by conventional ESE.

In this study, ESR-EB achieved a 100% complete resection rate, a finding that is consistent with prior reports^20^. The identical 100% complete resection rate observed in the ESE group, however, diverges from some earlier series^21,22^. This discrepancy is most likely attributable to the fact that all procedures were performed by a highly experienced team whose individual operators perform > 100 ESE procedures annually. Such expertise maximizes technical precision and minimizes the risk of margin-positive resections. Although the complete resection rates were equivalent, the ESR-EB procedure is considerably less complex, thereby conferring a theoretical advantage in reproducibility. In routine clinical practice, simpler techniques facilitate broader dissemination, particularly in resource-limited settings or regions where endoscopic proficiency is heterogeneous. Consequently, ESR-EB may offer a more readily adoptable alternative for achieving standardized, high-quality resection of gastric muscularis propria tumours.

The length of postoperative hospital stay did not differ between the two groups. This is most likely because neither group experienced major complications, and identical strategies were employed —namely, defect closure with endoscopic clips and comparable postprocedural nursing and rehabilitation protocols. Although ESR-EB conferred clear advantages in both overall and resection times, these benefits did not translate into earlier discharge, indicating that opportunities to accelerate recovery are common to both techniques and merit further optimization.

Previous studies have consistently shown that, among gastric subepithelial tumours ≤ 10 mm arising from the muscularis propria, GISTs are the most common histological diagnosis, followed by leiomyomas^2,4^. Our data corroborate this distribution: GISTs accounted for 32 of 54 tumours (59.26%) in the ESR-EB group and 27 of 54 tumours (50.00%) in the ESE group. These findings reinforce the rationale for resecting tumours in this size range. Because preoperative histological confirmation is often unattainable for ≤ 10 mm gastric muscularis propria tumours, a simple, effective and minimally invasive endoscopic approach—such as ESR-EB—may represent the most judicious therapeutic strategy.

Perforation was the most common intraoperative adverse event; it occurred in 30 patients (55.56%) in the ESR-EB group and in 21 patients (38.89%) in the ESE group. This difference did not reach statistical significance (*P* = 0.083). The higher perforation rate observed with ESR-EB is attributable to the technique itself: during negative-pressure suction, the entire gastric wall can be drawn into the transparent cap, increasing the risk of full-thickness entrapment. Nevertheless, all perforations in the ESR-EB group were closed endoscopically without sequelae. The brevity of ESR-EB resection limits the duration of perforation exposure, thus enabling rapid defect closure that reduces the incidence of pneumoperitoneum and peritoneal contamination^23–26^. In contrast, when perforation occurs during ESE, the defect remains open until tumour removal is complete, thus prolonging intraperitoneal air leakage, increasing the risk of infection and, importantly, degrading endoscopic visualization. These factors collectively increase the technical difficulty and time required for endoscopic suturing^27,28^.No gas-related complications occuerred in either group.Although the ESE has a long exposure time to perforation, the following two treatments can avoid serious pneumoperitoneum or postoperative peritoneal infection. The first reason is that the operator will reduce the gas input after the perforation is found. The second reason is that the carbon dioxide injection is used in the operation, and the gas is absorbed quickly after the operation.

Intraoperative bleeding occurred in 5 patients (9.26%) in the ESR-EB group, which was significantly fewer than the 18 patients (33.33%) in the ESE group (*P* = 0.002). The reason is that during ESE, mucosal incisions and tumour dissection are performed predominantly with a cutting current, which provides limited coagulation. When sizeable vessels are encountered, they are often transected before adequate haemostasis can be achieved, thereby increasing the likelihood of bleeding^29^. Conversely, ESR-EB involves an elastic band that first strangulates the tumour together with the overlying mucosa, submucosa, muscularis propria and serosa, effectively interrupting blood flow. Subsequent snare resection proceeds more slowly than the knife-based dissection used in ESE, thus allowing prolonged contact time for vessel coagulation. If a pure coagulation current is applied throughout the resection, the already low bleeding risk with ESR-EB could be reduced even further.

Postoperative prophylactic antibiotics were prescribed to 26 patients in the ESR-EB group (48.15%) and 17 in the ESE group (31.48%); this difference did not reach statistical significance (*P* = 0.077). The numerical excess in the ESR-EB group is likely driven by its higher intraoperative perforation rate, prompting clinicians to adopt a lower threshold for antibiotic prophylaxis. Nevertheless, the absence of a significant between-group difference underscores the need to scrutinize current prescribing patterns and to avoid unnecessary antimicrobial exposure. Previous work by Guohua Li et al.^30^ demonstrated a very low incidence of post-ESE bacteraemia, questioning the utility of routine prophylaxis. Comparable data for ESR-EB are lacking; prospective studies are therefore warranted to determine whether systematic antibiotic administration is justified after ESR-EB.

During the follow-up period, neither group experienced tumour recurrence or metastasis, indicating that both ESR-EB and ESE provide reliable long-term oncological control for patients with gastric muscularis propria tumours ≤ 10 mm. Nevertheless, given the relatively short duration of surveillance, extended follow-up is needed to confirm these findings and to detect any late recurrences or distant spread.

Several limitations should be acknowledged. First, this was a single-centre study with a modest sample size, which carries an inherent risk of selection bias; the generalisability of our findings therefore requires confirmation in larger, multicentre cohorts. Second, although propensity score matching was employed, residual confounding from unmeasured variables—such as comorbidities or lifestyle factors—cannot be excluded, and these factors may influence both procedural outcomes and recovery trajectories. Third, while the 2023 Chinese Expert Consensus recognizes EFTR as a standard treatment, we did not directly compare ESR-EB with EFTR. Fourth, In this study, endoscopic resection was performed by four different endoscopists. We could not avoid the differences in diagnosis and operation between endoscopists at different levels, but all endoscopists had more than 5 years of endoscopic experience and performed more than 100 independent endoscopic level 4 operations per year.We have added the above to the discussion.Finally, the relatively short follow-up period precludes definitive conclusions regarding long-term tumour recurrence, metastasis, or quality-of-life outcomes; extended surveillance is warranted to address these knowledge gaps.

In summary, both ESR-EB and ESE are effective and safe modalities for the resection of gastric muscularis propria tumours. However, ESR-EB is associated with a lower incidence of intraoperative bleeding and markedly shorter procedural and resection times. For gastric muscularis propria tumours ≤ 10 mm, ESR-EB represents a safer and more time-efficient endoscopic technique.

## Data Availability

Data is provided within the manuscript or supplementary information files.
